# Viral Vector Induction of CREB Expression in the Periaqueductal Gray Induces a Predator Stress-Like Pattern of Changes in pCREB Expression, Neuroplasticity, and Anxiety in Rodents

**DOI:** 10.1155/2009/904568

**Published:** 2009-04-01

**Authors:** Robert Adamec, Olivier Berton, Waleed Abdul Razek

**Affiliations:** ^1^Department of Psychology, Memorial University of Newfoundland, St. John's, Newfoundland, Canada A1B 3X9; ^2^Faculty of Behavioral Pharmacology, University of Pennsylvania, Room 2218 125, S 31ST (TRL) Philadelphia, PA 19104-3403, USA

## Abstract

Predator stress is lastingly anxiogenic. Phosphorylation of CREB to pCREB (phosphorylated cyclic AMP response element binding protein) is increased after predator stress in fear circuitry, including in the right lateral column of the PAG (periaqueductal gray). Predator stress also potentiates right but not left CeA-PAG (central amygdala-PAG) transmission up to 12 days after stress. The present study explored the functional significance of pCREB changes by increasing CREB expression in non-predator stressed rats through viral vectoring, and assessing the behavioral, electrophysiological and pCREB expression changes in comparison with handled and predator stressed controls. Increasing CREB expression in right PAG was anxiogenic in the elevated plus maze, had no effect on risk assessment, and increased acoustic startle response while delaying startle habituation. Potentiation of the right but not left CeA-PAG pathway was also observed. pCREB expression was slightly elevated in the right lateral column of the PAG, while the dorsal and ventral columns were not affected. The findings of this study suggest that by increasing CREB and pCREB in the right lateral PAG, it is possible to produce rats that exhibit behavioral, brain, and molecular changes that closely resemble those seen in predator stressed rats.

## 1. Introduction

Study of the neurobiology of long-lasting changes in affect occurring after stressful events is of interest, an interest heightened by the fact that fearful events may precipitate affective psychopathologies [1, 2]. In extreme cases, a single aversive experience may induce posttraumatic stress disorder (PTSD) [3, 4]. Animal models are useful to enhance understanding of the impact of stress on brain and behavior, permitting simulation of a human condition in a controlled setting allowing study of disorder development. Conditioned fear paradigms, behavior in unfamiliar situations that are fear or anxiety provoking, and more recently, predator stress, are all models used to understand the neurobiology of the impact of fearful events on affect.

Predator stress in our hands involves the unprotected exposure of a rat to a cat [5]. Predator stress may model aspects of PTSD for several reasons. First, predator stress has ecological validity due to the natural threat posed by the predatory nature of the stressor. Second, duration of anxiety-like effects in rats after predator stress, as a ratio of life span, is comparable to the DSM IV duration of psychopathology required for a diagnosis of chronic PTSD in humans. Third, predator stress has neurobiological face validity in that right amygdala and hippocampal circuitry are implicated in behavioral changes produced by predator stress, and these areas are consistent with brain areas thought to be involved in PTSD [6–9]. For example, brain imaging implicates hyperexcitability of the right amygdala in response to script-driven trauma reminders in the etiology of PTSD [10–14]. Fourth, parallel path analytic studies using data from Vietnam veterans suffering from PTSD and predator stressed rodents find that in both humans and rodents, features of the stressor predict the level of anxiety [6]. For example, in predator stressed animals, the more cat bites received, the higher the level of anxiety measured a week later. Finally, similar lasting changes in startle and habituation of startle are seen in both predator stressed rats and humans with PTSD [6, 15–18].

Predator stress is fear provoking and stressful [19–22]. Moreover, cat exposure produces long-lasting increases in rat anxiety-like behavior (ALB) [5, 23], with some behavioral changes lasting three weeks or longer [5, 6, 24]. Behavioral effects of predator stress have been evaluated in a number of tests including hole board, elevated plus maze (EPM), unconditioned acoustic startle, light/dark box, and social interaction. Anxiogenic effects of predator stress are NMDA receptor-dependent. Systemic administration of both competitive and non-competitive NMDA receptor antagonists 30 minutes before, but not 30 minutes after, predator stress prevents lasting changes in ALB [16, 25]. Moreover, local NMDA receptor block in the amygdala prevents predator stress-induced increases in ALB [26].

In addition to the behavioral changes, amygdala efferent and afferent neural transmission is altered after predator stress. Specifically, predator stress causes a long-lasting potentiation in neural transmission from the right amygdala (central nucleus-CeA) to the right lateral column of the periaqueductal gray (PAG), and from the hippocampus via the right ventral angular bundle (VAB) to the right basolateral amygdala (BLA) [9, 23, 27]. Moreover, potentiation in these pathways is NMDA receptor-dependent [7]. In addition, NMDA receptor antagonists produce anxiolytic-like effects when microinjected into the dorsolateral PAG [28, 29]. The PAG is also implicated in rodent ALB [30], and is activated by predator stress [31]. Together, these data suggest NMDA receptor-dependent long-term potentiation (LTP)-like change in amygdala afferent and efferent transmission following predator stress contribute to the lasting anxiogenic effects of cat exposure [7, 9, 16]. In support of this conclusion are the findings that amygdala afferent and efferent LTP-like changes are highly predictive of severity of change in ALB following predator stress [9, 23, 27].

Predator stress induced changes in ALB and amygdala neural transmission are accompanied by changes in phosphorylated cAMP response element binding protein (pCREB). Specifically, pCREB-like-immunoreactivity (lir) is elevated in the basomedial (BM), BLA, CeA, and lateral (La) amygdala after predator stress compared to control rats [32]. This is consistent with the elevation of pCREB-lir in the amygdala after forced swimming stress [33, 34], fear-conditioning in mice [35], retrieval of a cued-fear memory [36], and electric shock [37]. In addition to the amygdala, predator stress increases pCREB-lir in the right lateral column of the PAG (lPAG) [23].

As mentioned, NMDA receptor antagonism prior to predator stress blocks increases in ALB and potentiation of amygdala afferent and efferent neural transmission. Since phosphorylation of CREB may be regulated by NMDA receptors [38, 39] and pCREB-lir is increased after predator stress [23, 32], the question of whether NMDA receptor antagonism can block predator stress induced enhancement of pCREB-lir was recently tested. Blocking NMDA receptors with the competitive blocker, CPP, 30 minutes prior to predator stress, prevented stress induced increases in pCREB expression in amygdala, and right lPAG [40]. Of importance, the same dosing regime also blocks predator stress effects on affect and amygdala afferent and efferent transmission [7, 16, 25, 26].

Together these findings provide compelling evidence that predator stress induced increase in pCREB is an important contributor to the changes in brain and behavior of predator stressed rodents. The purpose of the present study was to directly manipulate CREB and pCREB expression to confirm this notion.

Local changes in gene expression in the brain can be achieved with viral vectoring as a method of delivering recombinant genes directly into neurons [41]. There are a variety of viral vectors available but several characteristics of the herpes simplex virus (HSV) make it an ideal candidate for this study. The non-toxic replication defective HSV vector is capable of infecting most mammalian differentiated cell types, it accepts very large inserts and has high efficiency in infecting neurons, being naturally neurotrophic [41, 42]. One of the earliest studies to utilize this method and apply it to rodent anxiety tests found that HSV vectored expression of CREB in the BLA increased behavioral measures of anxiety in both the open field test and the EPM, and enhanced cued fear conditioning [43].

The present study was designed to test the functional significance of pCREB changes within the right lateral column of the PAG. To do this we genetically induced increased expression of CREB in the right lPAG with HSV vectors and determined the effects of these manipulations on behavior and amygdala efferent transmission (CeA-lPAG). We transfected the neurons of the right PAG in an area where pCREB levels and CeA-PAG transmission are elevated after predator stress (see Adamec et al. [8, 23]).

## 2. Methods

### 2.1. Ethical Approval

The procedures involving animals reported in this paper were reviewed by the Institutional Animal Care Committee of Memorial University and found to be in compliance with the guidelines of the Canadian Council on Animal Care. Every effort was made to minimize pain and stress to the test subjects while using as few animals as possible.

### 2.2. Animals

Subjects were male hooded Long Evans rats (Charles River Canada). Rats were housed singly in clear polycarbonate cages measuring 46 cm × 24 cm × 20 cm for one week prior to any testing. During this week, rats were acclimatized to their cage, and handled. Handling involved picking up the rat and gently holding it on the forearm. Minimal pressure was used if the rat attempted to escape, and grip was released as soon as the rat became still. Rats were handled in the same room as their home cage for one minute each day during the week long adaptation period. Rats were given food and water *ad lib* and were exposed to a 12-hour light/dark cycle with lights on at seven a.m. The rats weighed approximately 200 g on arrival and between 230 and 280 g on the day of testing.

### 2.3. Groups

After lab adaptation and handling, the 12 subjects were randomly assigned to one of three groups of four. One group served as a handled control (Handled GFP) while another was predator stressed (Predator Stressed GFP). Both these groups were injected in the right lateral PAG (described in what follows) with the HSV-GFP vector before further treatment. This vector consisted of an HSV virus carrying a green fluorescent protein gene (GFP), a reporter used to visualize vector placement and virus induced gene expression. This injection also served to control for any effects that GFP per se might have. The third group was also handled (Handled CREB) and before further treatment received an injection in the right lateral PAG with an HSV-CREB vector. This vector included genes for both CREB and GFP. The GFP served as a reporter of gene expression, and the CREB gene elevated CREB levels in the target area.

It is recognized that a group size of four is small for behavioral studies of this nature. The small numbers were necessitated by the availability of the virus. The implications of the small group size are addressed further at the end of Section.

### 2.4. Surgical Microinfusion of Viral Vectors

Virus injections were done in the lateral column of the right PAG, where pCREB increases in predator stressed rats have been observed [23, 32, 40]. The injections involved lowering a sterile 25 gauge needle attached to a microliter syringe into the brain using a sterotaxically mounted microliter syringe holder. The coordinates for the microinfusion according to the atlas of Paxinos and Watson [44] were, 6.3 mm posterior to bregma, 0.5 mm lateral from the midline, and 5.5 mm below the skull. The injection of 0.5 *μ*L (in a concentration of 4.0 × 10^7^ infectious units/mL supplied from University of Texas South West Medical School) was given at a rate of 0.5 *μ*L per five minutes with the needle left in place for five minutes post injection. This dose and rate were derived from the experience with the vector of one of us (Berton). Moreover, in pilot studies with HSV-GFP, a 0.5 *μ*l injection at this rate produced GFP expression localized to the right lateral column of the PAG over an AP plane range of.7 mm at three days post injection, the time of maximal protein expression induced by this vector [43, 45].

Injections were performed under chloral hydrate anesthesia (400 mg/kg, IP) using aseptic technique. Preanaesthetic doses of atropine were given (1.2 mg/kg). Local anesthesia of wound edges was achieved with marcaine and epinephrine (2%) infusion and supplemented as needed. Holes in the skull were closed with sterile gel foam and sealed with sterile bone wax and scalp wounds sutured. Rats were kept warm under a lamp post surgery until they began to walk and groom, at which time they were returned to their home cage. Surgery took approximately one hour for each subject.

### 2.5. Cat Exposure and Handling Procedures

Three days after virus (HSV) injection, when viral expression is peaking [43, 45], rats were either handled or predator stressed. On the day of testing, predator stressed rats were exposed to the same adult cat as described elsewhere [5]. The cat exposure lasted 10 minutes and was videotaped to capture the activities of both the cat and the rat. The cat generally observed the rat at a distance with the intermittent approach and sniffing. On occasion, the cat would mildly attack the rat but no injuries were ever observed. At the end of the test, the rat was placed back into its home cage and left undisturbed. Rats in the other two groups whose treatment included only handling did not come into contact with the cat, cat odors or rats that had previously been exposed to cats. On the day of testing, rats in these groups were weighed and handled as previously mentioned for 1 min. After this handling period the rats were returned to their home cage and left undisturbed. Handled and predator stressed rats home cages were kept in separate rooms.

### 2.6. Behavioral Testing and Behavioral Measures

Four days after HSV injection and one day after treatment, ALB was measured in the hole board, EPM and startle tests. The hole board test took place just before the EPM as an independent test of activity and exploratory tendency [46].

#### 2.6.1. Hole Board and Elevated Plus Maze Testing and Measures

The hole board and EPM were constructed and used as described elsewhere [5]. The behavior of the rats in the hole board and EPM was videotaped remotely for later analysis. Rats were first placed in the hold board for 5 minutes. At the end of this time period they were transferred by gloved hand to the EPM for a further 5 minutes of testing. At the end of this testing period the rats were returned to their home cages.

Several measures of activity and exploration were taken while the rat was in the hole board. They included frequency of rearing (activity), and head dips, a measure of exploratory tendency scored when the rats placed its snout or head into a hole in the floor. Fecal boli deposited were also counted. A measure of thigmotaxis was time spent near the wall of the hole board. This measure was quantified as the rat having all four feet in the space between the holes for head dipping and the wall. Time spent in the center of the hold board was also recorded. A rat was considered to be in the middle when all four feet were in the center space defined by a square drawn through the four holes in the floor of the box.

In the EPM, exploration and activity were scored as the number of entries into the closed arms of the maze (closed arm entries). An entry was only recorded when the rat had all four feet inside one arm of the maze. Other measures of exploration included head dips, scored when a rat placed its snout or head over the side of an open arm, and rearing as a measure of activity. These behaviors were divided into three types: protected (rat had all four feet in closed arm for rearing or hindquarters in the closed arm for head dips), center (rat has all four feet in center of maze), and unprotected (rat has all four feet in an open arm). Time spent grooming was also recorded using the same three subdivisions.

Measures of anxiety-like behavior were also taken. Two measures assessed open arm exploration: ratio time and ratio entry. Ratio time was the time spent in the open arms of the maze divided by the total time spent in any arm of the maze. The smaller the ratio the less open arm exploration indicating a more “anxious” rat. Ratio entry was the number of entries into the open arms of the maze divided by the total entries into any arm of the maze. Again, the smaller the ratio, the less the open arm exploration experienced, the more “anxious” the rat.

Adamec and Shallow [5] were the first to adapt the concept of risk assessment to the EPM. This measure was scored when the rat poked its head and forepaws into an open arm of the maze while keeping its hindquarters in a closed arm. The frequency of risk assessment was measured and converted to relative risk assessment by dividing these frequencies by the time spent in the closed arms. Fecal boli deposited in the EPM were also counted.

#### 2.6.2. Startle Testing and Measures

Startle testing was conducted on the same day as the hole board and EPM. The startle response was determined using a standard startle chamber (San Diego Instruments). The apparatus was fitted with a 20.32 cm Plexiglass cylinder used to hold the animal during the test, as well as a speaker for producing the sound bursts. A piezoelectric transducer positioned below the cylinder detected motion of the animal in the cylinder. The output from this transducer was fed to a computer for sampling.

Prior to startle testing, animals were adapted to the apparatus for 10 minutes with a background white noise level of 60 dB. Then rats were subject to 40 trials (1/30 seconds) of 50 milliseconds bursts of 120 dB of white noise rising out of a background of 60 dB. Half the trials were delivered while the chamber was dark while the other half were delivered with an accompanying light (light intensity of 28 foot candles or 300 lux). The light trials were randomly interspersed among the dark trials. During the light trials, the lights would come on 2.95 seconds prior to the sound burst and remain on for the duration of the sound burst, terminating at sound offset (lights on for a total of 3 seconds). The chamber was in darkness between trials. A computer attached to the transducer recorded 40 samples of output. Samples included a 20 milliseconds baseline and 250 milliseconds sample after onset of the noise burst. Average transducer output just prior to noise burst was saved as a baseline (*V*_start_). The computer then found the maximal startle amplitude within each of the samples (*V*_max_). Both these measures were saved for later analysis. Peak startle amplitude was expressed as *V*_max_-*V*_start_ for analysis. At the end of the startle session the rats were returned to their home cages. The apparatus was washed between rats.

### 2.7. Electrophysiological Recording Procedure

Five days after HSV injection and two days after treatment, all rats were anaesthetized with urethane (1.5 g/kg) given in three divided doses separated by 10 minutes. Then the rats were placed in a sterotaxic instrument and injected under the scalp with marcaine (2% epinephrine) to locally anesthetize and reduce bleeding. The skull was exposed and holes drilled to permit stereotaxically guided insertion of stimulating electrodes into the central amygdala (CeA). Recording microelectrodes were placed into the PAG. Stimulating and recording electrode pairs were placed in both hemispheres. In addition, skull screws were placed over the olfactory bulb to serve as a ground and references. Stimulation electrodes were twisted bipolar stainless steel (0.125 mm in diameter, Plastics One) aimed at the CeA. Recording electrodes were stainless steel microelectrodes (1 *μ*m tip diameter, 0.6–1 MΩ, Frederick Haer) aimed at the PAG (verified coordinates appear in Table 1). Rats were placed in a shielded box for stimulating and recording experiments. Temperature was maintained between 36-37°C by a rectal thermistor connected to a digital thermometer and feedback control to a DC heating pad (Frederick Haer) under the rat. CeA was stimulated using a single biphasic constant current pulse (width .2 milliseconds) at 1/5 seconds over a range of intensities (.025–2.5 mA), 10 stimulations per intensity. Evoked potentials were sampled by computer and later analyzed from data stored on computer using DataWave software (see Adamec et al. [27] for further method details).

At the end of recording, rats were overdosed with Chloral Hydrate (1000 mg/mL, 1 mL, IP) and perfused with cold phosphate buffered saline and 4% Para-formaldehyde. Brains were extracted, sunk in 20% sucrose overnight at −4°C and then stored at −70°C. Subsequently brains were examined histologically for electrode locations, under green fluorescence microscopy to visualize GFP production and immunohistochemically to study pCREB expression.

#### 2.7.1. Electrophysiology Analysis Methods

The main measure of the size of the evoked potential was peak height (PH). The peak height at each intensity was taken by computer from field potential averages as illustrated in Figure 4. The raw PH at each intensity was expressed as a ratio of PH observed at threshold (see [23, 27]).

### 2.8. Immunocytochemistry

Thick frozen coronal sections (40 *μ*m) were cut from 5.8 to 6.8 mm posterior to bregma [44] to capture the same areas of the PAG studied in past predator stress experiments, and to capture the targets of virus injection and electrophysiological recording . Anterior-posterior (AP) plane location was determined by counting sections from the decussation of the anterior commissure (AP −0.26 from bregma, [44]) to the desired AP plane. This counting of sections allowed for an estimation of the AP plane position to the nearest 40 *μ*m during cutting. Every second section was saved, which provided 12 sections from each brain for processing. To ensure even distribution a multiple of three brains (one brain from each group) was cut and processed at the same time.

After sectioning, one section from each group was placed in a plastic tube with nylon covering at one end and then immersed in a plastic well containing phosphate-buffered saline (PBS). Each tube contained three sections, which were processed at the same time. The tubes were removed, blotted, submerged in a solution of normal goat serum and Triton X-100 and placed on a rocker for 1 hour. The sections were washed with PBS, blotted and incubated at −4°C for either 24 or 48 hours (reused antibody) in the primary phospho CREB antibody (Upstate/Chemicon). Consistent with past work [23, 32], a dilution of 1/500 for the primary antibody was used. After incubation, sections were washed again with PBS, blotted, and then immersed in the secondary biotinylated antibody (goat antirabbit) for 1 h. Sections were washed, blotted, and placed in the ABC (Vector Stain kit) solution for 1 h on a rocker. Finally, sections were washed with PBS for a third time, blotted and submerged in diaminobenzadine (DAB) solution for 5–25 min, monitoring for staining. Sections were then washed with PBS again, before mounting onto slides, dehydrated and cover slipped.

#### 2.8.1. Image Analysis (Densitometry)

Stained sections were analyzed blind to group assignment using image analysis software (MOKA software, Jandel). Hemispheres were measured separately. The PAG was divided into ventral, dorsal, and lateral areas to reflect the functional columnar organization described by Bandler and Depaulis [47]. This was done using the aqueduct of Sylvius as a guide. Horizontal lines were drawn from the top of the aqueduct to the outside edge of the PAG and from the bottom of the aqueduct to the outside edge of the PAG for both left and right hemispheres (see also [23]). The top sections were considered dorsal PAG, the middle sections were lateral PAG and the bottom sections were ventral PAG.

Raw pCREB lir densitometry data of each column in each hemisphere were converted to optical density (OD) units relative to the whole section. This was done by converting the raw PAG and raw whole section densitometry data to OD units via a calibrated step wedge. An image of the calibrated step wedge was taken at the same time as section images for each rat. Exponential fits of raw transmission values (*x*) to calibrated OD values were done by computer (Table Curve program, Jandel). All fits were good (all df adjusted *r* ^2^ > .9, *P* < .01). The exponential was then used to interpolate and convert raw transmission values to OD units. Analysis was performed on the ratio of average OD values in particular PAG areas to average OD values for the entire section.

## 3. Results

### 3.1. Anxiety-Like Behavior in the EPM

**Figure 1:**
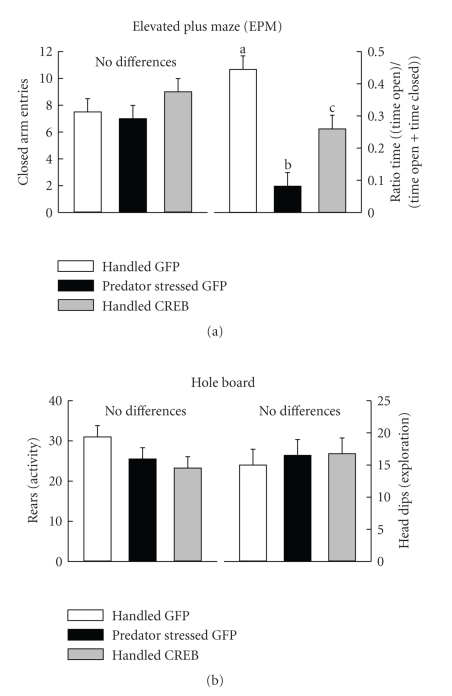
Plotted over groups are mean ± SEM of EPM and hole board behaviors. Means marked with a different letter differ from each other (*P* < .05). (a), shows frequency of closed arm entries (left) and ratio time (right) in the EPM. (b), shows the frequency of rears (left) and head dips (right) in the hole board test.

Groups differed in the measures of open arm exploration-ratio time and ratio entry (all *P* (2,9) ≥ 14.78, *P* < .002). Predator stress reduced ratio time and ratio entry in the EPM, consistent with many past studies (Figure 1, upper right panel, ratio time only is shown, ratio entry findings were very similar). Predator stress reduced open arm exploration (increased anxiety) the most relative to controls (Handled GFP). Injection of HSV-CREB in the right PAG alone was also anxiogenic in the EPM, reducing ratio time and entries in Handled-CREB rat to a level between Handled GFP and Predator Stressed GFP rats (Figure 1, Tukey Kramer test, *P* < .05).

**Figure 2:**
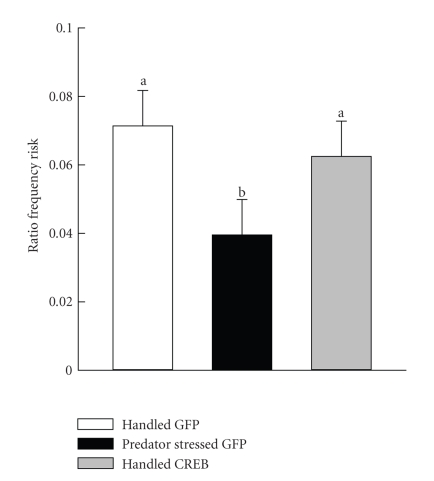
Plotted over groups are mean ± SEM of risk assessment behavior in the EPM. Means marked with a different letter differ from each other (*P* < .05).

With regard to ratio frequency of risk assessment, though there was no group effect (*F* (2,9) = 2.58, *P* < .13), a planned *t*-test contrasting the predator stressed group with the two handled groups combined (which did not differ) revealed that predator stress reduced risk assessment relative to both Handled groups (Figure 2; *t* (9) = 2.19, *P* < .029, 1 tailed). This finding of reduced risk assessment following predator stress is consistent with many previous studies.

### 3.2. Exploration and Activity in EPM and Hole Board

There were no differences between groups in closed arm entries (activity) in the EPM (Figure 1, (a) left panel). Similarly, groups did not differ in rears (activity) and head dips (exploration) in the hole board (Figure 1, (b) two panels). These data indicate that group differences in open arm exploration seen in the EPM are not the result of changes in activity or exploration.

### 3.3. Acoustic Startle Response

Startle in the light and dark trials did not differ so analyses across light and dark trials were combined.

#### 3.3.1. Startle Amplitude

**Figure 3:**
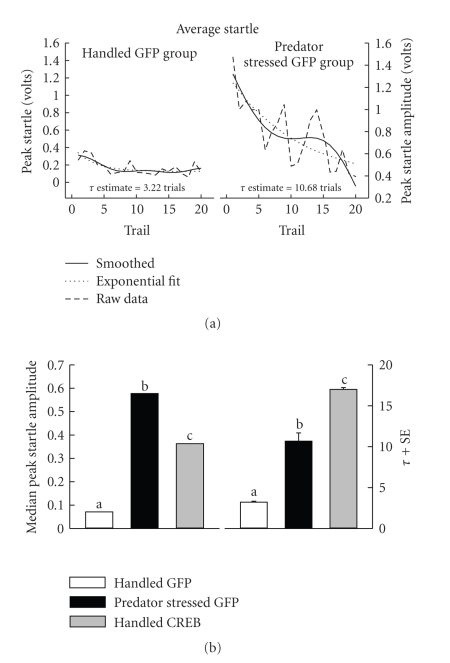
(a) shows example fits (solid line) to an FFT smoothed (20%) function (dashed line) of the means of peak startle amplitude (dotted line) over 20 trials for Handled GFP control (left) and predator stressed (right) rats. Plotted in (b) are median peak startle amplitudes (left) and *τ* ± SE (right), estimated from declining exponential functions, for rats in each experimental group. Medians and Tau values marked with a different letter differ from each other (*P* < .05).

Between groups startle data were not normally distributed (Omnibus Normality Test = 148.07, *P* < .0001). Therefore, Kruskal-Wallis one way non-parametric ANOVA on medians of peak startle amplitude over trials was used. Groups differed (*χ* ^2^ (2) = 119.90, *P* < .001). Planned comparisons (Kruskal-Wallis multiple comparison *z*-test *z* > 3.98, *P* < .01) revealed that predator stress increased startle over both handled groups (Figure 3, bottom left panel). Nevertheless startle amplitude of Handled CREB rats was also higher than Handled GFP, but lower than predator stressed animals (Figure 3, (b) left panel).

#### 3.3.2. Habituation of Acoustic Startle Response

Predator stress prolongs habituation to startle [6, 15, 16, 48]. Therefore, habituation to startle in the three groups was determined and compared. Exponential decline functions of the form

(1) $$y\;=\;y_{0}\;+\;ae^{-t/\tau }$$

were fit to the peak startle amplitude mean data from each group across 20 trials (combined light and dark startle trials) using Jandel table curve V 4.0. In (1), *y* and *y*_0_ are peak startle amplitude, *a* is a constant, *e* is the base of the natural logarithm, *t* is the trial number and *τ* is the trial constant, or the number of trials to decline to 37% of the maximal peak startle amplitude. To improve the fit, an FFT smoothing function provided by the program (20% FFT smooth) was applied. Care was taken to ensure the smoothing did not distort the data (Figure 3(a)). All fits were good (degrees of freedom adjusted *r* ^2^ > .84; all fits *F* (2,17) > 58.3, *P* < .001; *t* (38) ≥ 6.18, *P* < .01 for all *t*-tests of differences from zero of *τ*). The estimate of *τ* included a standard error of estimate. These standard errors were used to perform planned two tailed *t*-tests between groups using the different *τ* values (Figure 3(b), right panel). The pattern of the findings from this analysis was surprising. Both the Handled CREB and predator stressed group took significantly longer to habituate than Handled GFP controls. While this result was expected for the predator stressed group given previous work, the fact that the Handled CREB group took longer to habituate than the predator stressed group was uncharacteristic of the amplitude findings.

### 3.4. Electrophysiology

**Figure 4:**
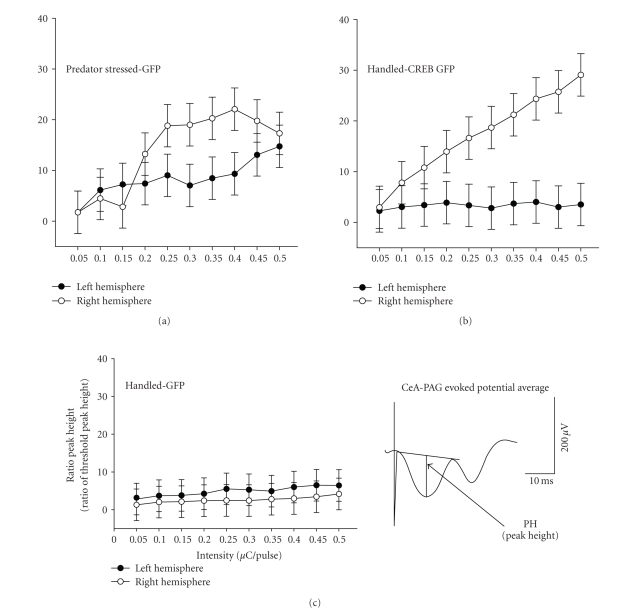
In the lower right is a computer average of a CeA-PAG evoked potential illustrating how peak height (PH) was measured by computer. Plotted in the graphs are means ± SEM of PH of CeA-PAG evoked potentials expressed as a ratio of threshold PH versus intensity of stimulation in *μ*C/pulse (calculated as intensity in *μ*A times pulse width in microsecond to take pulse width into the intensity measure). Means are plotted separately by group and within a group separately by hemisphere.

A three way ANOVA was done on ratio PH of the CeA-PAG evoked potential data. The factors examined were Group (Handled GFP control, predator stressed, and Handled CREB), Hemisphere (right and left) and Intensity of stimulation. There was a significant Group *x* Hemisphere *x* Intensity interaction (*F* (12,54) = 2.24, *P* < .04). The interaction is displayed in Figure 4. Intensity of stimulation was expressed in *μ*C (micro-coulombs) per pulse. All groups were stimulated using the same intensity series, so group differences cannot be attributed to difference in the intensity of stimulation.

Planned comparisons *t*-test mean contrasts were used to examine the interaction by comparing the three groups at each intensity in each hemisphere. All groups showed the same ratio PH values at intensity 1 in both hemispheres. Moreover, ratio PH in Handled GFP controls were equal in both hemispheres and unchanged over intensity of CeA stimulation (Figure 4(c)). Similarly, left hemisphere ratio PH of Handled CREB rats did not change over intensity and did not differ from ratio PH in right or left hemisphere of Handled GFP controls. In contrast right hemisphere ratio PH of Handled CREB rats rose over intensity (*t*(54) = 5.61, *P* < .01, comparing intensity 1 and 10) and differed from their own left hemisphere ratio PH over intensities 2–10 (all *t*(54) > 2.80, *P* < .05; Figure 4(b), top right panel). Therefore, CREB injection per se selectively potentiated right hemisphere CeA-PAG evoked potentials relative to the left hemisphere and relative to Handled GFP controls, which did not differ from Handled CREB rats in the left hemisphere.

As might be expected from previous work, predator stress potentiated right and left hemisphere CeA-PAG evoked potentials (Figure 4(a), upper left panel). Ratio PH in left and right hemispheres rose over intensity (all *t*(54) > 4.68, *P* < .01, comparing intensity 1 and 10). However, right hemisphere response exceeded the left at intensities 4–9 (all *t*(54) > 2.09, *P* < .05). This suggests that left hemisphere potentiation in predator stressed rats was fading relative to right hemisphere potentiation two days after treatment. Nevertheless, predator stress potentiated left CeA-PAG ratio PH over that seen in the left hemisphere of Handled CREB rats or in the left or right hemispheres of Handled GFP control rats, in that left ratio PH of predator stressed rats exceeded left ratio PH of Handled CREB rats (and left and right ratio PH of Handled GFP control) rats at intensities 3, 5, 9-10 (all *t*(54) > 2.04, *P* < .05).

Comparing right hemisphere ratio PH of Handled CREB and predator stress rats suggests nearly equal potentiation. Groups did not differ at intensities 1-2 and 4–8, but Handled CREB ratio PH did exceed that of predator stressed at intensities 3, 9 and 10 (all *t*(54) > 2.15, *P* < .05). Therefore right PAG CREB injection per se is as effective, or even more effective, than predator stress in potentiating right CeA-PAG evoked potentials.

### 3.5. Histological Verification of Electrode and Cannula Placements

Tips of stimulating and recording electrodes were visualized microscopically from tissue sections and plotted onto rat atlas sections [44]. Rats from all three groups had correctly placed electrodes, allowing the use of each subject for data analysis. Two way ANOVAs were done examining group and hemisphere factors with separate analyses for the coordinates of each plane (AP, lateral and vertical) for each electrode target. Lateral and vertical coordinates were taken from the atlas sections while AP plane was calculated from section number. No group, hemisphere, or groups *x* hemisphere interactions were observed. The CeA stimulating electrodes were correctly placed in the medial central nucleus while the recording electrodes were in the lateral columns of the right and left PAG. Average location of tips for both the stimulating and recording electrodes appear in Table 1. Verification of cannula placement was completed in much the same way, average coordinates appear in Table 2. Furthermore, the absolute distance from the recording electrode was very small (Table 2) indicating that electrophysiological recordings were taken from a position close to viral injection.

### 3.6. pCREB lir Immunohistochemistry Densitometry Analysis

**Figure 5:**
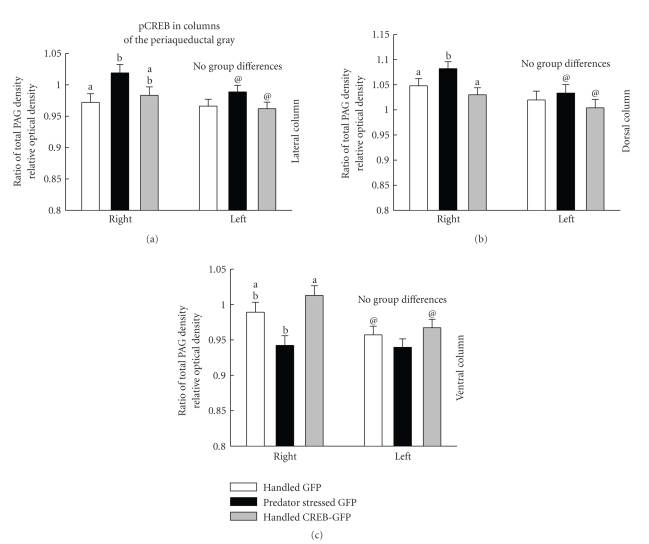
Mean ± SEM relative optical density units (PAG optical density units divided by total PAG section optical density units) in all three columns for all experimental groups are plotted. The left side of each panel displays data from the right hemisphere while the right side of the panel illustrates left hemisphere data. For a given column, means marked with the same letter do not differ, but differ from those with different letters, while means marked with two letters do not differ from means marked with either of the letters (Tukey-Kramer tests, *P* < .05). Means marked with “@” show a within group difference between hemispheres (*P* < .05 1 tailed test).

Relative OD data were analyzed separately for each of the three columns in the PAG. The lateral column was of primary interest since this was the area where CREB protein expression was enhanced (Figure 5(a), top left panel). A one way ANOVA of right hemisphere data revealed a significant difference between the groups (*F* (2,41) = 3.30, *P* < .05). In contrast, groups did not differ in the left hemisphere (*F* (2,41) = 1.88, *P* < .17). Predator stressed rats had significantly more pCREB lir than Handled GFP controls with the Handled CREB rats falling in between these two groups, differing from neither (Tukey-Kramer Test, *P* < .05). The mean of pCREB lir in Handled CREB rats measured here at 5 days post HSV injection is likely an underestimate of its value at peak expression of CREB, which occurs at three days after HSV injection, when treatments occurred (stress or handling), and which fades thereafter [43].

One tailed *t*-tests were used to compare within groups across hemispheres based on the prediction that right column pCREB-lir would be increased in predator stressed rats based on previous findings, and on the prediction that increased CREB expression in Handled CREB rats would increase pCREB-lir. Both the predator stressed and Handled CREB rats exhibited more pCREB lir in the right over the left (all *t*, *P* < .04, 1 tailed), whereas there were no hemisphere differences in the Handled GFP control group.

Data from the dorsal column of the PAG were analyzed in the same way with somewhat differing results (Figure 5(b), top right panel). A one way ANOVA of right hemisphere data revealed a significant difference between groups (*F* (2,41) = 3.66, *P* < .04) while the left hemisphere again showed no group difference (*F* (2,41) = 0.74, *P* < .49). Comparison of the groups in the right hemisphere revealed that the predator stressed rats showed elevated pCREB lir which was greater than the Handled groups which did not differ (Tukey-Kramer tests, *P* < .05). Furthermore, comparison of groups across the two hemispheres revealed that, like the lateral column, both the stressed and Handled CREB group had elevated pCREB lir in the right hemisphere as compared to the left (all *t*, *P* < .04 *t* tailed) with the Handled GFP control group again showing no difference between hemispheres.

Expression of pCREB in the ventral column of the PAG presented another pattern of results (Figure 5(c), bottom panel). A one way ANOVA in the right hemisphere revealed a significant difference between groups (*F* (2,41) = 6.93, *P* < .003). In this case however, the stressed rats had significantly lower pCREB expression than Handled CREB rats with the Handled GFP control group falling in between, differing from neither (Tukey-Kramer tests, *P* < .05). Much like the other two columns, no difference was seen between groups in the left hemisphere (*F* (2,41) = 1.36, *P* < .28). Comparisons within groups across hemispheres showed that both the Handled GFP control and Handled CREB rats had increased pCREB in the right over the left hemisphere (all *t*, *P* < .01), while there was no hemisphere difference in predator stressed rats. These ventral column results mirror previous findings with the exception of the hemisphere differences [40]. The fact that the Handled CREB group did not differ from the Handled GFP controls indicates that CREB may not be having an effect in this column. This also suggests that regional differences in the pathways controlling phosphorylation of CREB may be dependent on predator stress.

### 3.7. Visualization of GFP

**Figure 6:**
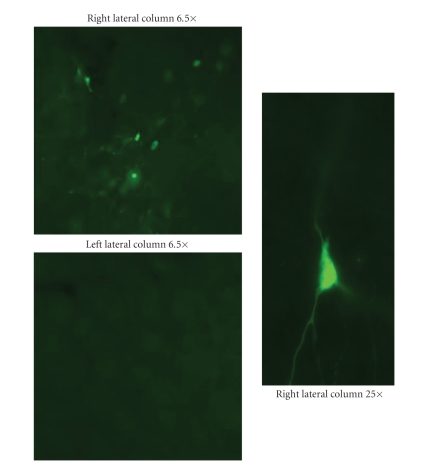
Depicted in the figure are fluorescence photomicrographs illustrating green florescent protein (GFP) localized in the right lateral column of the periaqueductal gray (PAG) 5 days after HSV injection in the right lateral PAG. Magnifications are 6.5 and 25 times (×).

Verification of gene expression was achieved by examining all PAG sections taken for green flourescence as evidence of expression of the reporter GFP. Green flourescence in the right PAG verified gene expression of GFP occurred after HSV injection in the vicinity of the injection cannulas and PAG recording electrodes (Figure 6, e.g., five days after HSV injection). Since flourescence ranges from cannula to PAG electrodes, one can derive a sense of the AP plane range of gene expression from PAG electrode position relative to cannulas. Referring to Table 2, evidence of gene expression five days post HSV injection appears over a range of ±.28 ±.034 mm (mean ± SEM) from the cannula in the AP plane. This represents a range nearly as extensive in previous pilot work which found that at the time of peak gene expression (three days post HSV injection), GFP expression was localized to the right lateral column of the PAG over an AP plane range of ±.35 mm from the cannula.

### 3.8. Power Associated with Significant Results

Given the small n of groups, power (*α* =.05) of all significant findings was calculated. Significant behavioral and electrophysiological findings all had power values in excess of.90. Power associated with pCREB expression analyses varied with column of the PAG, ranging from .82 to .91 in the dorsal and ventral columns to a reduced power for the lateral column results of .60.

The power of a test depends on the value of the type I error (here *α* = .05), the sample size, the standard deviation, and the magnitude of the effect being tested reflected here in magnitude of mean differences. Most findings appear quite robust with power values in excess of .80, suggesting robust effects of predator stress and virally induced CREB expression on brain and behavior. The reduced power for the lateral column pCREB findings suggests a fading effect in this column.

## 4. Discussion

The primary purpose of this study was to examine the functional significance of pCREB changes within the right lateral column of PAG. This was accomplished by genetically inducing an increased expression of CREB, through viral vectoring, and determining the behavioral, electrophysiological and pCREB expression changes in comparison to predator stressed and Handled GFP control rats.

### 4.1. Behavioral Effects of Viral Vectoring CREB

Viral vectoring to induce CREB expression in the right lateral column of the PAG produced behavioral effects resembling those seen in predator stressed rats. Handled CREB rats showed increased open arm avoidance in the EPM (decreased ratio time and entry) as compared to Handled GFP controls. However, predator stress was even more effective, increasing open arm avoidance over that seen in Handled CREB rats. Despite, this graded change in anxiety between groups, measures of activity and exploration in the plus maze or hole board did not differ (Figure 1). This pattern of results suggests changes in open arm exploration (anxiety) in EPM are not due to changes in activity or exploratory tendencies, consistent with previous findings using predator stressed rats in similar testing situations [5, 9, 15, 23, 25, 32].

The ability of CREB per se to increase open arm avoidance in the absence of any predator stress is a remarkable finding. It suggests a direct role for CREB and possibly pCREB expression [32] in behavioral changes produced by stress. Predator stress likely induces CREB signaling change, and then behavioral changes via NMDA receptor activation in the PAG [7, 23, 25, 40]. In the present study, stress effects were mimicked by bypassing the NMDA receptor activation and directly activating CREB mediated processes.

Not all effects of predator stress were mimicked by PAG CREB induction, however. Normally predator stress reduces ratio frequency risk assessment in an NMDA receptor-dependent manner [5, 7, 16, 25, 26]. While predator stress in the present study also reduced risk assessment, the risk assessment of Handled CREB rats was unaffected and did not differ from Handled GFP controls (Figure 2). The lack of a predator stress type response in the Handled CREB rats suggests that increasing CREB expression in PAG may not be the only factor that mediates suppression of risk assessment, or alternatively may only affect some EPM behaviors. Other necessary factors at play could include changes in amygdala pCREB expression and potentiation of ventral hippocampal to BLA transmission, both of which follow predator stress [32]. In addition, risk assessment changes produced by predator stress are highly predicted by right hemisphere changes in transmission in both CeA-PAG and hippocampal to BLA pathways [9]. Since only PAG was manipulated in Handled CREB rats, it is likely these other factors were not engaged, but were engaged in predator stressed rats. Perhaps changes in risk assessment require all changes to occur. A change in hippocampal spatial information transfer to BLA might make sense, since risk assessment is described as a form of sampling the immediate environment for potential threats [49]. Other possible reasons include the following. Handled CREB rats were more anxious than Handled GFP controls in EPM, but their level of anxiety was not as great as predator stressed rats. Greater levels of anxiety may be associated with less risk assessment [49], and so the more anxious predator stressed rats displayed reduced risk assessment. Further testing will be required to decide between these possibilities.

Handled CREB rats also had elevated median peak startle amplitude in comparison to the Handled GFP control group. Moreover, the predator stressed group showed startle amplitudes that surpassed those of the Handled CREB rats. This graded response of enhanced startle over groups is reminiscent of open arm avoidance in the EPM, and supports the notion that inducing CREB expression per se induces an anxious state which is milder than that produced by predator stress. Reasons for the milder effects of direct PAG manipulation in comparison to predator stress may parallel those raised above to explain risk assessment discrepancies. Finally, the enhancement of startle amplitude in predator stressed rats is consistent with past studies [6, 23, 26, 27].

Predator stress also reliably decreases rate of habituation of the acoustic startle response [15, 16, 48]. Present data are consistent with these findings in that predator stressed rats took significantly longer to habituate than Handled GFP controls (Figure 3). This replication furthers the validity of predator stress as a model of hyperarousal aspects of PTSD, since delayed habituation to startle is also observed in PTSD patients [50–53].

Surprisingly, the Handled CREB group took even longer than the predator stressed rats to habituate to startle. This finding implicates CREB dependent mechanisms in delay of startle habituation, which are likely NMDA receptor-dependent, given that CPP administered 30 minutes prior to predator stress blocks delay of startle habituation as well as increased right lateral PAG pCREB expression [16, 40]. However, this finding also suggests some difference in mechanisms of induction of neural changes by CREB in PAG underlying enhanced startle amplitude and delay of habituation. Delay in startle habituation has been observed in the absence of increased startle amplitude making it likely that different neural circuits/mechanisms mediate changes in these two responses to acoustic startle [7, 16]. Additionally, recent studies suggest that separate portions of the CeA-PAG pathway mediate the stress induced changes in startle amplitude and startle habituation [7]. Another possible explanation could be the following. Though NMDA receptor-dependent potentiation of efferent transmission from amygdala to PAG mediates increases in startle amplitude [9, 23, 26], it is homosynaptic depression in brain stem startle pathways that underlies habituation [54], and direct CREB expression in PAG more powerfully engaged such depression than predator stress per second.

### 4.2. Effects of Viral Vectoring CREB on CeA-PAG Transmission

A fascinating finding was that viral vectoring of CREB induced a potentiation of the CeA-PAG pathway in the right hemisphere (Figure 4) analogous to that seen after predator stress. Moreover, potentiation in this group was restricted to the same hemisphere as injection. In fact the evoked potentials in the left hemisphere of the Handled CREB rats did not differ from those observed in Handled GFP controls. This implies that any behavioral changes observed in this group can be attributed to the change in transmission due to CREB induction in the right hemisphere.

In past studies, CeA-PAG potentiation by predator stress has been shown to be NMDA receptor-dependent. CPP administration prior to predator stress blocks both anxiogenic effects and and CeA-PAG potentiation [7, 16]. Moreover, given that predator stress induces NMDA receptor-dependent right PAG pCREB expression, it has also been suggested that long lasting right CeA-PAG pathway potentiation is dependent on pCREB expression [7, 40]. Present findings in Handled CREB rats support this hypothesis.

The present study also adds new data on the time course of CeA-PAG pathway potentiation in predator stressed rats. Current results show that, as expected, predator stressed rats exhibited potentiation in the right CeA-PAG pathway two days after predator stress (Figure 4), complementing those studies that have replicated this finding at 1, 9 and up to 12 days post predator stress [9, 23, 32]. A novel finding was the fading, but still present, potentiation in the left CeA-PAG of predator stressed rats. The presence of potentiation in the left hemisphere adds to previous studies showing left CeA-PAG one day after predator stress [27], but fading completely by 9 days [7]. Present findings suggest a left hemisphere potentiation lasting at least two days.

The presence of bilateral CeA-PAG pathway potentiation in predator stressed rats and the unilateral induced right CeA-PAG pathway potentiation in Handled CREB rats at the time of anxiety testing may account for some of the differences in open arm avoidance, risk assessment, and startle response between groups. This especially concerns the absence of reduced risk assessment in the Handled CREB group, since NMDA block in the left dorsolateral amygdala 30 minutes prior to predator stress prevents stress effects on risk assessment [26]. Moreover path analysis suggest that changes in open arm exploration and risk assessment may depend on bihemispheric changes in limbic transmission in the early stages after predator stress [27].

Long lasting potentiation in the right CeA-PAG pathway by predator stress has been suggested to reflect some, but not all, of the anxiogenic neuroplastic changes after predator stress [9, 23]. Taken together present findings lend strong support to this view.

### 4.3. Effects of Viral Vectoring CREB on pCREB lir

Given that predator stress increases pCREB lir selectively in the right lateral column of the PAG, and that CeA-PAG potentiation persists longer in the right hemisphere, it has been suggested that increased production of pCREB underlies right CeA-PAG potentiation. Furthermore, degree of pCREB expression and right CeA-PAG potentiation correlate highly with the same measures of the predator stress experience suggesting a strong relationship between these two phenomena [23].

In the present study, densitometry analysis revealed a right over left lateral PAG increase in pCREB lir in both Handled CREB and predator stressed groups. Thus increasing CREB expression directly and genetically in the right lateral PAG also increased pCREB in a pattern similar to predator stress in a group which had not been predator stressed. Moreover, in Handled CREB rats, the increase of pCREB in the right but not left lateral column of the PAG is consistent with potentiation in the right but not left CeA-PAG pathway in this group. The fact that pCREB expression in the right lateral column in the Handled CREB group was intermediate, neither differing from the predator stressed group nor the Handled GFP controls, is consistent with their milder than predator stressed rats increase in anxiety in the EPM and acoustic startle tests. Taken together, these results support the suggestion that elevated pCREB leads to neuroplastic changes that induce right CeA-PAG potentiation and increased anxiety [23, 32].

This conclusion must be tempered by the reduced power associated with lateral column significant findings. The reduced power here likely reflects a reduced effect evidenced in the small mean differences encountered in the analyses. As pointed out above (Section 3.6) the mean of pCREB lir in Handled CREB rats measured at 5 days post HSV injection is likely an underestimate of its value at peak expression of CREB, which occurs at three days after HSV injection, when treatments occurred (stress or handling), and which fades thereafter [43]. Moreover, effects of predator stress on pCREB expression are evident at 20 minutes post stress and fade thereafter (20 and unpublished observations). Since transient NMDA receptor block prevents predator stress effects on brain and behavior and suppresses pCREB expression [7, 25, 26, 40], it is likely that changes in brain and behavior depend on immediate effects of increased pCREB expression, which in this study would have likely begun before the time of pCREB measurement. Further studies examining CREB and pCREB expression in lateral PAG at 1–3 days post HSV injection are required to clarify present findings.

Present findings mirror those seen in previous work with respect to the lateral column of the PAG. However, the dorsal column results in comparison require greater interpretation. The pattern of dorsal column pCREB changes stand in contrast to findings that predator stress alone does not alter pCREB lir in this column when measured 20 minutes after predator stress [23, 40]. In the current study predator stressed rats had elevated pCREB expression in the right dorsal PAG, while the two Handled groups had lower and similar levels of expression two days after treatment (Figure 5). A right over left hemisphere expression effect was observed in both the predator stressed and Handled CREB groups, similar to the lateral column. The fact that the right exceeds the left in the Handled CREB rats suggests that right lateral column pCREB enhancement may have spread to the dorsal column, but not enough to differ from the Handled GFP control. Other explanations include a potential leak up the cannula tract or the possibility that this is a function of CREB induction, since the predator stressed group demonstrated similar effects though more pronounced. The increase of right over left pCREB expression in predator stressed rats suggests that the EPM is having an effect on the dorsal column up to 24 hours later. This extends previous findings which showed that dorsal column pCREB was elevated bilaterally in predator stressed rats 20–25 minutes after exposure to the EPM which took place 7 days after predator stress [55]. Previous and present findings differ, however, in that in the present study, there was no pCREB increase over control in the left hemisphere in predator stressed rats. This suggests that an increased time interval between the predator stress experience and EPM testing may allow for left hemisphere pCREB levels to increase. Conversely, in the present study 24 hours elapsed between EPM testing and pCREB testing. Perhaps left dorsal column pCREB expression faded over this time interval. Further research into time course of pCREB changes following predator stress and EPM exposure seems warranted.

Though lateral and dorsal column findings are somewhat in line with previous wok, the results of the ventral column are not. In the present study pCREB expression in predator stressed rats was decreased in comparison to both Handled groups in the right hemisphere, and right and left hemisphere expression did not differ in predator stressed rats. Moreover, Handled groups displayed increased pCREB expression in right over left hemispheres (Figure 5). There are discrepancies and similarities with previous work examining pCREB expression 20 minutes after handling or predator stress. Previous work showed no differences in pCREB expression between predator stressed and handled controls in ventral PAG of both hemispheres, with right hemisphere expression elevated over the left [23]. Perhaps differences in time of sampling pCREB expression accounts for the discrepancies between past and present findings, since pCREB in the present study was measured two days after treatment.

If decreases of pCREB expression in ventral PAG are normally delayed after predator stress (for which we have preliminary evidence, unpublished data), then present findings suggest such decreases are independent of enhanced pCREB expression in lateral PAG induced by direct genetic induction at least. If increase in lateral column and decrease in ventral column pCREB expression parallel enhancement and suppression of normal functioning, then one might suspect a shifting of defensive response bias toward avoidance of threatening stimuli and away from a relaxed immobility, along the lines of functional columnar differences in the dorsolateral and ventral PAG described by Depaulis and Bandler [47]. Further time course studies of shifting defensive response bias following predator stress seem warranted.

### 4.4. Summary and Conclusions

In summary, the present study demonstrated that directly inducing CREB (and pCREB) expression in the right lateral PAG reproduced behavioral, brain, and molecular changes that closely resemble those seen in predator stressed rats. These findings suggest increased CREB (and perhaps pCREB) expression in the lateral PAG is at least sufficient to produce brain and behavioral changes normally induced by a brief predator stress. Moreover, similar effects of inducing CREB expression in basolateral amygdala on EPM anxiety at least, have been reported by Wallace et al. [43]. Together these data support the idea that the CREB-pCREB pathways in the right lateral PAG, and perhaps amygdala, are important entry level molecular paths to lasting anxiogenic effects of predator stress. To the extent that predator stress models some aspects of PTSD, present finding point to CREB and pCREB pathways as possible new therapeutic targets.

## Figures and Tables

**Table 1:** Mean (and SEM) of electrode coordinates averaged over group and hemisphere.

Brain area	Mean	SEM
CeA AP	2.37	0.036
CeA Lateral	3.98	0.037
CeA Vertical	7.92	0.027

PAG AP	6.1	0.061
PAG Lateral	0.33	0.02
PAG Vertical	5.62	0.034

AP: Anterior-posterior plane (mm posterior to Bregma); Lateral: lateral plane (mm lateral to mid line); Vertical: vertical plane (mm below Bregma) ; CeA: central amygdala; PAG: periaqueductal gray

**Table 2:** Mean (and SEM) of PAG cannula coordinates and in relation to PAG electrodes.

Right cannula	Mean	SEM	Mean absolute distance (mm)	SEM
			from the right PAG electrode	
PAG AP	6.13	0.068	0.28	0.034
PAG Lateral	0.36	0.032	0.09	0.026
PAG Vertical	5.62	0.04	0.11	0.028

AP: Anterior-Posterior Plane (mm posterior to Bregma) Lateral: Lateral Plane (mm lateral to mid line) Vertical: Vertical Plane (mm below Bregma) PAG: Periaqueductal Gray
